# “A small change can make a huge difference”: teachers’ perceived roles, barriers, and strategies in tackling loneliness in schools

**DOI:** 10.3389/fpsyg.2026.1766329

**Published:** 2026-02-23

**Authors:** Yixuan Zheng, Charlotte Bagnall, Caroline Bond, Elizabeth Birchinall, Pamela Qualter

**Affiliations:** 1 College of Teacher Education, Taishan University, Tai’an, China; 2 Manchester Institute of Education, University of Manchester, Manchester, United Kingdom

**Keywords:** attachment, cross-cultural comparison, pupil loneliness, teacher perspectives, thematic analysis

## Abstract

Teachers, as key attachment adults in the school environment where children and adolescents spend substantial time, play a crucial supportive role in pupil’s experiences of loneliness. This study captures teachers’ perspectives on pupil loneliness through qualitative interviews with 16 primary and secondary school teachers (8 from England and 8 from China), covering pupils ages 6–18 years. Thematic analysis of those interviews uncovered four themes: Teachers as Key Attachment Figures in School Life, Teachers’ Relational Work in Addressing the Social Dimensions of Loneliness, Professional and Institutional Barriers, and Teachers within a Multi-layered Attachment Ecology. Understanding teachers’ perspectives helps to bridge the gap between research and practical application. The findings reveal various barriers that limit teachers’ ability to alleviate pupil loneliness effectively; using socioecological model, we see those range from intrapersonal challenges, such as limited knowledge and reliance on rigid methods, to external factors like inadequate school resources and results-driven school policies. Despite those challenges, teachers wanted to support pupils who reported loneliness. Building on teachers’ insights, we recommend addressing loneliness in schools by (1) integrating loneliness-specific interventions within a broader mental health framework, and (2) fostering a proactive, whole-school supportive climate. We outline a framework with four core elements—Knowledge Development, Social and Emotional Skills, Communication and Collaboration, and Observation and Adaptation—that should be incorporated into loneliness-focused teacher training programs. Including teachers from two countries allowed for a cross-cultural comparison of support strategies, underscoring the need for adaptable, universal frameworks and highlighting the importance of cultural sensitivity when developing interventions and sharing insights across cultural contexts.

## 1 Introduction

Loneliness has been recognized as a public health concern (Lancet, 2023). A recent report from the World Health Organization (2025) estimates that 1 in 6 people worldwide is affected by loneliness. It is distinct from aloneness (Goossens et al., 2009) and prevalent among school-aged children and adolescents (Qualter et al., 2021; Surkalim et al., 2022) due to the critical developmental stages they are navigating (Williams et al., 2017). Given the amount of time that young people spend in school, it makes sense to consider whether schools are a natural context in which to provide intervention/prevention opportunities for loneliness. Schools are where learning happens, but they are also where friendships form and socio-emotional ability develops (Roeser et al., 2000). At the same time, schools can be settings where pupils experience stress or comparison with peers (Murray-Harvey and Slee, 2007). This study explores how teachers - the main adult figures pupils interact with daily - perceive their role in supporting pupils who report loneliness, and reflect the current school-based interventions. Furthermore, schools function within the cultural and political climates of their respective countries, which means feelings of loneliness in the school context and the support provided by teachers are influenced by broader societal systems and norms. By interviewing teachers from both England and China, this study aims to explore how cultural norms influence teachers’ approaches to supporting pupils experiencing loneliness.

### 1.1 Loneliness among children and adolescents

Loneliness is understood as an unpleasant emotional experience stemming from a lack of meaningful social connections (Perlman and Peplau, 1982). Loneliness is a subjective feeling, distinct from being alone: an individual may be physically alone without feeling lonely, or feel lonely despite being surrounded by others (Mauri et al., 2024). Attachment theory offers a framework for explaining why some individuals are more vulnerable to loneliness than others. According to Bowlby (1969), humans are born with an attachment behavioral system that motivates them to seek proximity to significant others—attachment figures—particularly when facing real or symbolic threats. When individuals perceive that such figures are unavailable or unresponsive, insecure attachment patterns may emerge (Bowlby, 1982, 1988). These patterns, including avoidant or anxious attachment, have been associated with higher levels of loneliness (Goldman et al., 2025; Mikulincer et al., 2021). In this sense, loneliness in childhood is not simply about being alone; it reflects a felt absence of reliable attachment and emotional connection, accompanied by a longing for closeness that is unmet (Qualter and Munn, 2002; Cassidy and Berlin, 1999). Vulnerability may be heightened during adolescence, a period in which young people begin to shift attachment needs from parents to new figures, including peers and extrafamilial caregivers like teachers, yet may not have fully formed these new attachment relationships securely (Al-Yagon et al., 2016; Goossens et al., 1998). During this transitional phase, feelings of loneliness often increase (Ribeiro et al., 2025).

Loneliness during children and adolescence is not always a passing feeling, but can become a chronic condition that disrupts a young person’s developmental trajectory. It can negatively affect academic performance, hinder the development of social skills, and diminish overall quality of life (Arslan, 2020; Berguno et al., 2004; Jefferson et al., 2023a). Furthermore, youth loneliness has been linked to both mental and physical health challenges (Christiansen et al., 2021; Hards et al., 2021; Zheng et al., 2024a; Zheng et al., 2025). Studies also suggest that loneliness experienced at a young age is strongly associated with loneliness at other life stages (Bartels et al., 2008; Matthews et al., 2019). Given the long-term impact of loneliness, it is crucial to focus on addressing this issue during these formative years.

### 1.2 The role of teacher in pupil loneliness

Although Bowlby’s original formulation of attachment theory focused on primary caregivers within the family, later research (Bowlby, 1973) extended the framework to include other significant adults who provide reliable emotional support in young people’s everyday lives (Pianta, 1999; Al-Yagon and Mikulincer, 2006). Within the school environment, teachers are context-specific attachment figures, “stronger and wiser” adults (Bowlby, 1982), and are often the most influential non-familial figures in youth social worlds. They may act as safe havens in times of need and as secure bases from which pupils explore, learn, and develop skills (Cassidy, 2008). Beyond classroom instruction, teachers act as key facilitators of emotion management, helping pupils navigate loneliness and preventing transient feelings of loneliness from becoming chronic. A recent meta-analysis reported a moderate negative association between the quality of teacher-student relationships (TSRs) and loneliness (Zheng et al., 2024b), suggesting that strong, affectionate bonds with teachers can buffer against loneliness by providing pupils with a sense of security and belonging. In addition, teachers function as socializers within the classroom (Galanaki and Vassilopoulou, 2007), shaping peer interactions, guiding constructive conflict resolution, and helping pupils build positive group relationships.

Empirical evidence also suggests that pupils do not always perceive teachers as well-positioned to support loneliness (Verity et al., 2022). This may reflect structural limitations of teacher–student relationships, which lack the exclusivity and long-term continuity characteristic of “fully-fledged” attachment bonds (Verschueren and Koomen, 2012). At the same time, the fact that loneliness is not directly observable raises an important question: how can teachers identify loneliness when it may not manifest in overt behaviors? In the current study, we seek to explore teachers’ perspectives on this issue by listening to their voices and experiences, paying attention to how they interpret their responsibilities, how they attempt to recognize it in practice, and what motivates or constrains their involvement. We also explore whether any reluctance to intervene in loneliness stems from a lack of skills, resources, or understanding, or if they do not view it as part of their role, focusing instead on academic responsibilities.

### 1.3 School-based interventions for loneliness

Young people develop within organized and dynamic systems that include multiple proximal and distal layers of influence (Pianta et al., 2003). The dyadic teacher–pupil interactions, as one of these proximal processes, are situated within broader systems, most immediately, the school environment. Building on work conducted by Qualter (2003), a narrative review by Jefferson et al. (2023b) identified several individual risk factors, including low resilience, low self-esteem, and skill deficits, that could be targeted through focused interventions within schools. Besides individual factors, the school environment itself could function as a protective context, shaping how relationships are formed, maintained, and experienced. Evidence from multilevel modelling indicated that differences between European schools account for approximately 20% of the variation in pupils’ reported feelings of loneliness (Schnepf et al., 2023). In particular, supportive school climates foster positive relationships with teachers and promote cooperation and companionship among peers, enhancing pupils’ sense of attachment and commitment toward their school community (Zheng et al., 2024a).

Reviews of school-based mental health interventions acknowledge that teachers are valuable partners with school mental health professionals, and effective delivery of interventions would not be possible without their collaboration (Banwell et al., 2021; Franklin et al., 2012). Understanding teachers’ perspectives can provide valuable insights into the contextual factors that influence the implementation of mental health practices. By capturing these real-world perspectives, research can identify feasible strategies and adapt evidence-based interventions to fit everyday classroom contexts, thereby helping to bridge the gap between theoretical knowledge and practical application in schools (Graham et al., 2011). Moreover, schools are increasingly positioned as part of a broader, multi-agency approach to adolescent mental health and wellbeing (O’Reilly et al., 2018). In this context, teachers serve as natural implementers of school-based interventions. However, there is limited understanding of teachers’ views on existing school-based methods for addressing loneliness, and their readiness to engage in interventions specifically targeting loneliness. By exploring teachers’ experiences, the current study aims to enhance our understanding of how schools can better support pupils experiencing loneliness and develop more effective, teacher-informed interventions.

### 1.4 Cross-cultural perspectives on teacher support and loneliness

The nature of teacher-student interactions is influenced by cultural backgrounds and expectations. Hofstede (1986) indicated that teachers in individualist societies are encouraged to be strictly impartial, whereas in collectivist societies, teachers are often expected to offer preferential treatment to specific pupils. China, for example, is a highly collectivist and large power distance culture dominated by traditional Confucian ideals. In China, the teaching profession is often regarded as “the most respected profession” (Hofstede, 1986). Teachers in this context play a crucial role in fostering a communal sense of identity and support, which mitigates feelings of loneliness among pupils (Chen, 2010). In contrast, the teacher-student relationship in England is less about hierarchical respect and more about mutual engagement and personal development, aligning with the individualist ethos of the UK (Hofstede, 1983). By comparing teachers’ voices across these two cultural contexts, we aim to understand how cultural norms and values shape teachers’ approaches to interaction and support, as well as their views on support from other stakeholders. Grasping these cultural nuances is crucial for developing effective interventions tailored to the specific needs of different cultural groups.

### 1.5 Study purpose

Through semi-structured interviews with teachers from England and China, the current study investigated teachers’ perceived roles in supporting lonely pupils, and their perceptions of current school-based interventions. Using Thematic Analysis, we revealed key themes that provide deep insights into the data. The study aims to capture multiple facets of teachers’ views, addressing the following research questions (RQs):

*RQ1*: What are teachers’ perceptions on how to support lonely pupils and how can these support strategies be improved in practice?

*RQ2*: Hoes does culture influence teachers’ approaches to supporting loneliness, particularly in a comparison between teachers from England and China?

## 2 Methods

### 2.1 Participants

In this study, we interviewed 16 teachers from primary and secondary mainstream schools, with an equal representation from England (8 teachers) and China (8 teachers). The purposive maximum variation sampling method (Robinson, 2014) ensured diversity in factors such as age, educational level, and subject expertise, capturing a range of perspectives within the teaching profession. Participants also varied in their years of teaching experience, further enriching the data with insights from both early career and experienced teachers.

Gender distribution among the participants was carefully aligned with the real-world teaching profession, which is female dominated (Department of Education, 2023; UNESCO, 2020), resulting in a 1:3 ratio of male to female participants. Table 1 provides detailed information of the participants’ demographic characteristics.

**Table 1:** Participants’ demographic characteristics.

Teachers background								Pupils background			
Teacher No.	Country	Gender	School type	Teaching subject	Grade	Teaching experience	Mode of interview	Age (in years)	Gender proportion	Ethnicity	Socioeconomic background
BP1F1	UK	Female	Primary school	All subjects, mainly arts and well being	Year2	Experienced teacher	Online	6–7	Balanced	Mixed ethnic background	High
BP1M1	UK	Male	Primary school	All subjects, mainly science	Year2-4	Experienced teacher	Online	6–9	Balanced	Mixed ethnic background	Average
BP1F2	UK	Female	Primary school	All subjects	Year2	Early career teacher	Online	6–7	More boys	Mixed ethnic background	NA
BP1F3	UK	Female	Primary school	All subjects	Year 5	Experienced teacher	In-person	9–10	Balanced	Predominantly white	Low
BS1M1	UK	Male	Secondary school	Physical education (PE)	Year 7–13	Experienced teacher	In-person	11–18	Mixed	Mixed ethnic background	Average
BS1F1	UK	Female	Secondary school	Psychology	Year 12–13	Experienced teacher	In-person	16–18	More girls	Mixed ethnic background	Average
BS1F2	UK	Female	Secondary school	Business, Health and social care	Year 7–13	Experienced teacher	Online	11–18	Mixed	Mixed ethnic background	Low
BS1F3	UK	Female	Secondary school	German, Spanish	Year 7–11	Experienced teacher	Online	11–16	More boys	Predominantly white	High
CP1F1	China	Female	Primary school	Chinese, Morality and Law	Year 2	Experienced teacher	In-person	7	More girls	All Han ethnicity	Average
CP1F2	China	Female	Primary school	Math	Year 5	Experienced teacher	In-person	10–11	More boys	All Han ethnicity	Low
CP1M1	China	Male	Primary school	Science, Morality and Law	Year 3, 5	Experienced teacher	In-person	8, 10	More boys	All Han ethnicity	Low
CP1F3	China	Female	Primary school	English	Year 6	Early career teacher	Online	11–12	More boys	Predominantly Han ethnicity	High
CS1M1	China	Male	Secondary school	Physical education (PE)	Year 9	Experienced teacher	In-person	14–15	Balanced	Predominantly Han ethnicity	Average
CS1F1	China	Female	Secondary school	Biology	Year 11	Early career teacher	Online	16–17	More girls	All Han ethnicity	Average
CS1F2	China	Female	Secondary school	Chinese	Year 8	Early career teacher	Online	13–14	More boys	All Han ethnicity	High
CS1F3	China	Female	Secondary school	Chinese	Year 12	Early career teacher	Online	17–18	Balanced	All Han ethnicity	High

NA = not available.

### 2.2 School, cultural, and linguistic contexts in England and China

In England, schooling is commonly framed as serving both academic and personal developmental aims. Alongside subject learning, schools are expected to support pupils’ social, emotional, and mental wellbeing, for example through Personal, Social, Health and Economic (PSHE) education, pastoral systems, and whole-school wellbeing initiatives. Teachers often hold formal or informal pastoral responsibilities (e.g., form tutors or mentors), and emotional support is widely recognized as part of the professional role. At the same time, schools operate within a strong accountability framework, with inspection and performance measures placing sustained emphasis on academic outcomes.

In China, the education system places a more explicit and central emphasis on academic attainment, particularly in secondary education where high-stakes examinations—most notably the *gaokao* (national university entrance examination)—structure pupils’ educational trajectories and school priorities. Teachers are primarily evaluated on pupils’ academic performance, and classroom instruction is often oriented toward examination preparation. Nevertheless, teachers—especially *banzhuren* (class teachers)—are also responsible for pupils’ moral education, daily management, and communication with families, resulting in a role that combines instructional authority with broad supervisory and caregiving duties. In addition, some schools employ psychology teachers who provide professional mental health support; however, these roles are often limited in number and scope.

Beyond broader cultural orientations—such as the contrast between individualistic and collectivist traditions discussed in the Introduction—language also forms an important contextual layer. In English, “loneliness” is generally understood as a negative emotional state associated with social disconnection and psychological distress. In Chinese, however, the commonly used term *gudu* encompasses a broader range of meanings. While these linguistic distinctions are relevant for understanding how loneliness may be perceived and addressed by teachers in each context, the present study does not assume fixed meanings. Instead, it recognizes that teachers’ interpretations of loneliness are shaped through their professional experiences and everyday interactions with pupils, which are explored empirically through the interview data.

### 2.3 Data collection

Semi-structured interviews, guided by an interview protocol (see Supplementary material), enabled the first author (YZ) to explore teachers’ perceived roles, needs, and challenges in supporting pupils experiencing loneliness. The original interview guide was developed in English and translated into Mandarin for use with Chinese teachers. The translation was refined following a pilot interview with a native Chinese-speaking teacher, ensuring that the language and framing were easily understood and contextually appropriate. Interviews were conducted either online or face-to-face, depending on participants’ preferences and availability, and lasted an average of 43 min. All sessions were audio-recorded with participants’ consent and subsequently transcribed verbatim. Interviews conducted in Mandarin were translated into English by trained undergraduate research assistants, and the first author (YZ) performed back-translation checks to ensure semantic accuracy and fidelity to the original meanings. Interview topics included teachers’ attitudes towards school-based intervention and their strategies for addressing it in school settings. For example, questions asked were: “How good effective do you think you are at helping a lonely pupil?” and “Is the teacher’s role clearly or vaguely defined towards lonely pupils in your school?”

### 2.4 Data analysis

Reflexive Thematic Analysis (Braun and Clarke, 2022) was used to analyze the data, with an inductive data-driven coding approach to ensure themes were generated directly from the data, thereby minimizing potential researcher bias from pre-existing theoretical frameworks and assumptions. Initial coding was performed by YZ and CBa on a subset of transcripts. Key themes regarding teachers’ perceived needs, supporting strategies, and barriers were developed, refined, and discussed to ensure alignment with the study’s aims.

In conducting the cross-cultural analysis, we systematically examined both similarities and differences at the semantic level from transcripts of teachers in China and England, focusing on how they describe and respond to pupil loneliness. This approach is grounded in the principle that language reflects implicit “cultural scripts” within a society (Wierzbicka, 1994). By mapping these semantic patterns, we identified areas of convergence and divergence across cultural contexts, shedding light on shared themes as well as culturally specific nuances in how loneliness amongst pupils is supported.

### 2.5 Ethical considerations

The study received ethical approval from The University of Manchester’s Research Ethics Committee (ref: 2023–17656-30812). Before participating in the interviews, the teachers received a participant information sheet and provided written informed consent by completing and signing the study consent form. The documents were developed in English. To ensure comparability, we also prepared a Chinese version of all documents (e.g., PIS, consent form, interview guide) that mirrors the UK version. Prior to the interviews, the interviewer restated pertinent information with participants, addressed any inquiries, and informed them that they had 1 week to withdraw their own data. To ensure confidentiality, pseudonyms are used below in the reporting, and interview data was pseudonymized through the assignment of participant IDs (e.g., CP1S1).

### 2.6 Trustworthiness

To enhance trustworthiness, the research process incorporated member checking, translation accuracy checks, and an audit trail including memos and a reflexive journal. Researcher bias was minimized through consensus-based coding discussions and interrater reliability checks, with regular meetings to ensure the consistency of interpretations across the research team.

## 3 Results

**Figure 1:**
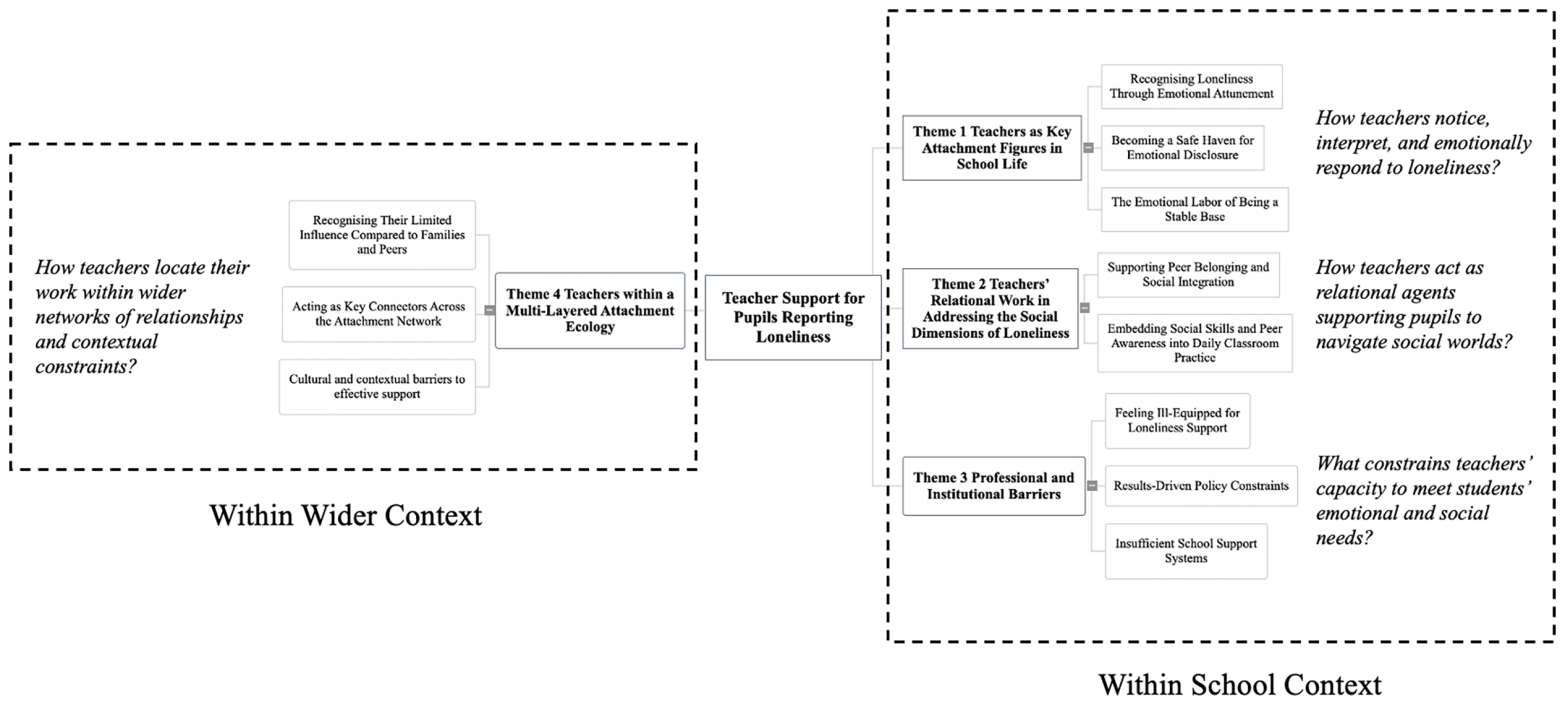
Interconnections between themes.

Using Inductive Thematic Analysis, we identified four overarching themes that were organized according to the primary aspect of loneliness-related support they captured: emotional and attachment processes (Theme 1: *Teachers as Key Attachment Figures in School Life*), relational and social practices (Theme 2: *Teachers’ Relational Work in Addressing the Social Dimensions of Loneliness*), professional and institutional constraints (Theme 3: *Professional and Institutional Barriers*), and the broader ecological positioning of teachers’ roles within and beyond school (Theme 4: *Teachers within a Multi-Layered Attachment Ecology*). Figure 1 illustrates the interconnections of those themes. While attachment theory informed the interpretive framing and organization of themes at a later analytic stage, all codes, subthemes, and themes were generated inductively from participants’ accounts rather than being pre-specified by theory.

For RQ1, we explored practical perspectives, delving into teachers’ perceptions of how to support lonely pupils within the school context and their role in the broader environment, and examining their views on other support providers and the potential barriers hindering such practices. We identified four main themes and eleven subthemes. Regarding RQ2, we compared semantic similarities and differences between teachers from England and Chinese teacher groups. The main themes that emerged are displayed in the center, with culture-specific insights organized under each respective cultural group, as illustrated in Table 2. Below, we present the themes and sub-themes, along with relevant quotes from participants. To simplify comparison, quotes from participants from England are marked as “B,” while those from Chinese participants are labelled as “C”; “P” to the participants from primary school, and “S” to secondary school participants.

**Table 2:** Thematic map outlining specific cultural differences, specific to teachers in China and England (in brackets).

Teachers in China	Themes and subthemes	Teachers in England
	*Theme 1: Teachers as key attachment figures in school life*	
	1a Recognizing loneliness through emotional attunemnet	
*[building closer personal connections]*	1b Becoming a safe haven for emotional disclosure	*[building emotional connections]*
	1c The emotional labor of being a stable base	
	*Theme 2: Teachers’ relational work in addressing the social dimensions of loneliness*	
*[saving “face”]*	2a Supporting peer belonging and social integration	*[avoiding awkward or uncomfortable]*
*[weekly class meeting]*	2b Embedding social skills and peer awareness into daily classroom practice	*[PSHE curriculum]*
	*Theme 3: Professional and institutional barriers*	
	3a Feeling Ill-equipped for loneliness support	
*[exam-centered prioritization]*	3b Results-driven policy constraints	*[continuous accountability pressure]*
*[class teacher]*	3c Insufficient school support systems	*[pastoral team]*
	*Theme 4: Teachers within a multi-layered attachment ecology*	
*[support from grandparents]* *[community support]*	4a Recognizing their limited influence compared to families and peers	*[support from siblings]*
*[teachers move from the school to the home environment]*	4b Acting as key connectors across the attachment network	*[parents move from the home to the school environment]*
*[the sensitivity to adapting educational concepts in different cultural contexts]*	4c Cultural and contextual barriers to effective support	

### 3.1 Theme 1: Teachers as key attachment figures in school life

Focusing on how teachers notice, interpret, and emotionally respond to loneliness, we identified three subthemes: *1a recognizing loneliness through emotional attunement, 1b Becoming a Safe Haven for Emotional Disclosure,* and *1c The emotional labor of being a stable base*.

#### 3.1.1 1a. Recognizing loneliness through emotional attunement

Teachers described recognizing loneliness as a process of emotional attunement rather than a one-off act of identification. Teachers emphasized empathy, approachability, and sensitivity to pupils’ emotional states as central to this process. One Chinese secondary school teacher noted: “I’m pretty empathetic, so I’m usually very tuned in to how he’s feeling” (CSF2), while an English teacher similarly stated: “You need to be approachable and be a good listener” (BPF1). For some teachers, emotional attunement also involved creating spaces in which pupils felt able to express their feelings: “The first thing to do is to try every means possible to let the pupil express what he or she wants to express, or their hidden feelings, so that we can then help solve these problems” (CPM1). At the same time, teachers across both contexts acknowledged that loneliness is a subjective emotional experience, which introduces uncertainty into attempts at recognition. As one English secondary school teacher reflected, although quietness might signal loneliness, “it could be other issues as well that causes them to be quiet” (BSF1). Teachers also highlighted the difficulty of maintaining closely attuned to individual pupils’ emotional worlds in crowded classrooms: “We try our best, but there are so many children” (BPF2); “It is inevitable that sometimes signs are missed earlier on…it’s really difficult to make sure you are meeting the needs of every single child in your room” (BPF1).

Because teachers cannot directly access pupils’ internal emotional experiences, they described relying on indirect and observable cues as a way of inferring loneliness. These included patterns of peer interaction: “When they got into pairs, he was often one of the last to be picked” (BPF1). Teachers also picked up on nonverbal cues such as seating choices and “facial expressions” (BPM1). Noticing subtle behavioral changes was also important: “They’ve changed the way they behave, or you notice them not being with others” (BSF2). Chinese teachers additionally referenced academic performance and family background as contextual signals that informed their interpretations, such as noticing a “poorer academic score” (CSF1) or insights gained from “understanding his family background” (CPM1). Importantly, teachers themselves recognized the limitations of relying on observable indicators to identify a deeply internal emotional state. Several acknowledged that loneliness can remain “under the radar” (BPF3), particularly when behavioral cues are subtle or when pupils “mask” their feelings in front of teachers (CSF3).

#### 3.1.2 1b. Becoming a safe haven for emotional disclosure

Recognizing loneliness is only a first step. Teachers across both contexts highlighted the need to sustain emotional safety in order for pupils to feel secure enough to reveal and work through their feelings: “They feel very comfortable talking to you about their worries, talking to you about their emotions. They know that they’ll be listened to” (BPF2). Simple acts of kindness—such as offering words of encouragement (“praise every step forward, no matter how tiny”; CSM1), a smile (BPF3), a supportive gesture (“lower our posture, that is, squat down and talk to the child”; CPF1), or a small, personalized acts of care (“little rewards and things that she was interested in”; BSF2)— were described as integral to this process. As one Chinese secondary school teacher explained, “Children like this… may feel warm, like sunshine” (CSF1). Similarly, a Chinese primary school teacher emphasized that “You need to make him feel that you care about him from the bottom of your heart” (CPF3).

Teachers also described strengthening emotional safety through shared experiences and personal stories. One Chinese teacher explained, “A teacher plays the role of an actor: ‘I experienced the same kind of thing when I was a child’, and then he will gradually approach you from his inner side” (CPF1). An English teacher similarly noted, “I showed him a picture when I went to the dentist rather than just being the teacher who stands up and tells them something, you are actually a real, whole person with similar experiences as well” (BPM1). Compared with their English counterparts, Chinese teachers more often described forming closer personal bonds with pupils, including engaging in home visits (CSF3, CSM1) and offering support that extended into pupils’ daily lives: “I knew about her family’s difficulties from the beginning, so I donated my daughter’s clothes to her” (CPF1), reflecting a deeper, more intensive approach to care. They emphasized a nurturing role, particularly in primary school settings, where female teachers were expected to be “mother-like” and male teachers “father-like” (CPF2, CPM1).

#### 3.1.3 1c. The emotional labor of being a stable base

Teachers described the role of being a stable emotional base for lonely pupils as involving sustained emotional labor. Maintaining emotional consistency—“being very consistent with how we are, how we handle our emotions, how we communicate with them” (BSM1)—was seen as essential, yet emotionally demanding. A Chinese primary school teacher reflected on the difficulty of fully separating personal emotional states from interactions with lonely pupils: “Sometimes, I brought my own emotions to the situation at first” (CPM1). Teachers also noted that heavy workloads and time pressures intensified this strain, not simply as practical constraints, but as factors that depleted their emotional capacity and made it harder to respond with flexibility and care. As one English teacher explained, although they knew “the kind of person you need to be,” emotional and situational pressures sometimes meant that “the strategies you use are limited” (BPF1).

Teachers further emphasized that emotional labor involved resisting the urge to intervene quickly or superficially. One teacher cautioned, “When you are facing a lonely child, you must not rush for success. It will go in the opposite direction” (CPF3). This need to remain emotionally steady and adaptable—even when progress was slow—was described as taxing but necessary. This teacher’s personal journey described a shift from initial frustration to deeper emotional commitment: “I was really troubled at first. It gave me a lot of additional work. But gradually I realized, wow, she is really the child I like. I think I will regret it if she continues like this, and then I will sincerely give her a lot of my own tolerance, love, and support” (CPF3).

### 3.2 Theme 2: Teachers’ relational work in addressing the social dimensions of loneliness

In examining how teachers act as relational agents supporting pupils to navigate social worlds, we identified two subthemes: *2a Supporting Peer Belonging and Social Integration*, and *2b Embedding Social and Emotional Learning into Daily Classroom Practice*.

#### 3.2.1 2a. Supporting peer belonging and social integration

Teachers across both countries described playing an active role in helping pupils navigate the social world of the classroom. They highlighted how the everyday physical and social organization of classroom life—such as seating arrangements, grouping patterns, and activity structures—shapes pupils’ opportunities to form connections. As one English primary school teacher stated, “if the teacher moves the groups around to suit different activities, then the children… learn how to work with people that they know and those they do not get on with so well” (BPM1). Strategic grouping and careful movement of pupils were viewed as practical tools for broadening social networks and reducing the risk of isolation: “Sometimes, there are lonely children whom others are reluctant to include. In these situations, the teacher must take the initiative to approach the child, ask them who they’d like to join, and foster an atmosphere for successful group integration” (CSM1).

Beyond these visible structural adjustments, teachers emphasized the importance of fostering connections in less obvious ways. Instead of overtly pairing pupils to “fix” loneliness, several preferred to engineer conditions in which relationships could form naturally. As one English primary school teacher noted, “if you can create situations where they are coming together more naturally and able to find peers that way that they can connect with, then that works much better than being able to force a situation” (BSF2). Similarly, a Chinese primary school teacher described deliberately positioning a lonely pupil within a socially supportive peer group: “I intentionally placed the girl with the class’s most inclusive group. In it, a caring classmate acts like an older sister who will look out for her, and there are no harsh or mocking peers” (CPF3). This reflects a shared belief that true integration should be gently scaffolded: “I think there has to be a balance because you do not want to be overdoing it…that you make them possibly feel anxious about coming to class” (BSF1).

Cultural differences shaped how this subtlety was understood and enacted. Chinese teachers emphasized the importance of “saving face” (Mian Zi), a core social value in East Asian cultural contexts that prioritizes maintaining social dignity, moral standing, and harmonious relationships within the group. As one Chinese primary school teacher explained, “My first consideration was saving face for the child. Well, even he was in first grade” (CPM1). Teachers were particularly attentive to the risk that overt support might result in labeling, loss of status, or peer stigmatization. On the other hand, teachers in England more often framed their concerns around avoiding situations that might feel “awkward” or “uncomfortable” for the pupil (BSM1). Rather than focusing primarily on social reputation within the group, these teachers emphasized individual emotional comfort and psychological wellbeing.

#### 3.2.2 2b. Embedding social skills and peer awareness into daily classroom practice

Teachers in both countries suggested going beyond basic knowledge transfer to incorporate social skills and relational competencies into everyday teaching. As one Chinese teacher noted, “We can also teach them some additional knowledge about social life” (CSF1). Importantly, this relational learning was often directed not only toward pupils experiencing loneliness, but also toward their peers: “We teach them to engage with that person and ask them, would you like to play with us? And just in general, making sure that, if you see somebody that’s not kind of involved in a conversation, try and involve them” (BPF2). However, opportunities to embed such relational learning varied across subjects and teaching contexts. Some secondary school teachers described disciplinary constraints that limited explicit discussion of social relationships: “Perhaps there is not much room for us to play in our subject (Biology). But in subjects like History or Chinese, there is more scope” (CSF1).

Beyond subject-specific teaching, some teachers included the topic of loneliness and peer relationships into designated curricular or routine spaces. In England, one teacher included loneliness into the PSHE (Personal, Social, Health, and Economic) curriculum: “We did a lesson on loneliness. We teach them what loneliness is, what it looks like to experience it as a person, and what to do if you think somebody is lonely or feeling lonely” (BPF1). In contrast, Chinese teachers described the absence of a specific formal curriculum focused on loneliness, but emphasized making use of weekly classroom meetings (a routine teacher-led session covering moral, social, and collective issues). One Chinese teacher remarked, “I think it’s just about making full use of the class meeting time. We have a different theme every week that covers a variety of topics” (CPF2).

### 3.3 Theme 3: Professional and institutional barriers

Teachers highlighted the importance of school life for young people, emphasizing that schools are not just transient places: “School is their whole world… if they are coming in every day without having built relationships or friends to talk to and interact with, I think that can make them feel really lonely” (BPF1). Teachers described various school support methods, such as the pastoral system in England, peer-led group activities, and annual mental health assessments. Despite these efforts, several barriers hinder effective teacher support and school intervention, summarized below.

#### 3.3.1 3a. Feeling ill-equipped for loneliness support

Echoing insights from theme1a, teachers often feel ill-equipped with professional knowledge specific to loneliness, relying instead on personal awareness, accumulated experience, and moment-to-moment judgement in their interactions with pupils. As one Chinese primary school teacher reflected, “I think there are no specific skills, it’s just exploration. Well, everyone is just exploring” (CPF3). This lack of specialist knowledge sometimes resulted in uncertainty about whether their responses aligned with pupils’ actual needs. As one English secondary school teacher noted, “Some teachers’ way of helping them might not be the way pupils think” (BSF2). Further, beyond conceptual understanding, teachers were also eager for actionable steps that are grounded in research and translated to what they can used in the everyday classroom: “I would like to acquire more of the kind of pedagogical side linked to research…things that you could do that would work. More like practical suggestions” (BPF1).

This reflected the absence of dedicated teacher training focused specifically on loneliness. As one English teacher stated, “I have done training about children’s wellbeing generally, but nothing about loneliness specifically” (BPM1). Similarly, a Chinese teacher observed, “To be honest, it seems that there is currently no such project” (CSF2). This lack of focused training leaves teachers feeling underprepared to identify and respond to the unique challenges of pupil loneliness. This lack of targeted professional development left teachers feeling underprepared to recognize the nuanced forms of loneliness or to engage in sensitive conversations about pupils’ emotional experiences, often accompanied by self-doubt. One teacher candidly reflected, “I just say what I think. I do not know if that’s okay” (CSF2).

#### 3.3.2 3b. Results-driven policy constraints

Teachers across both contexts described a persistent tension between policy-level commitments to child-centered education and the realities of working within increasingly results-driven school systems. Although schools often highlighted values around wellbeing and holistic development, teachers felt that academic performance indicators routinely took precedence over pupils’ social and emotional needs. As one English teacher reflected, “I’m not seeing schools do this in the extreme that they are at the moment, the standard strives, I would call it, is very high and we value at the moment politically and knowledge-based, fact-driven curriculum” (BPF3). Another teacher similarly noted that “the Department for Education and Ofsted are very result driven. And because of that, I think there’s a lot of time pressure on focusing on the academic side of things, and I worry that potentially more children who aren’t getting helped” (BPM1). In the Chinese context, this results-oriented climate was described as particularly pronounced in upper secondary school, where preparation for the *gaokao* (the national university entrance examination) dominated school life. As one teacher explained, “Most of their lives are actually about studying… At this stage, my aims are very utilitarian. What I want for them is to gain skills that translate directly into higher marks” (CSF1).

While the policy mechanisms differed—continuous accountability pressures in England or exam-centered prioritization in China—teachers in both systems described limited space for proactive attention to early, preventative work around loneliness. Teachers described being forced into reactive roles, responding to difficulties only once they became visible or disruptive, rather than addressing emerging emotional needs proactively. One teacher noted, “It has not happened yet, and the staff has not been able to allocate some resources to these children” (CSF2), suggesting that support was often triggered by crisis rather than by early signs of vulnerability. An English teacher similarly expressed concerns that “I think teachers are increasingly becoming almost firefighters…batting away the big behaviors and you become quite reactive rather than proactive” (BPF3).

#### 3.3.3 3c. Insufficient school support systems

Efforts to support pupils effectively were constrained by funding limitations, large school sizes, and insufficient staffing, which together limited teachers’ capacity to provide sustained social and emotional support (BPF3). many teachers highlighted the practical difficulty of monitoring pupils’ social experiences in overcrowded environments. As one English teacher explained, “there’s 180 children out on the playground. So, it’s hard, obviously, to keep an eye on every child and make sure everybody is playing with somebody” (BPF2). Although some schools do have specialized support staff focusing on pupils’ mental health and wellbeing, these resources are stretched thin. One teacher explained, “There are only two psychology teachers (school-based staff with formal responsibility for delivering mental health education and providing psychological support to pupils) in our school, so they are actually very busy, because we have a total of 40 classes” (CSF3). Alongside these specialist roles, both systems relied heavily on non-specialist pastoral staff embedded in everyday school life. In England, teachers referred to form teachers, mentors, and trained teaching assistants, while in China this responsibility largely fell to the *banzhuren* (class teacher). However, these roles were often accompanied by extensive academic and administrative workloads. As one Chinese teacher explained, “I do not have additional time on school days. We go to school at 7:30 in the morning and there is after-school service, which means teachers stay in the school until 6 p.m. It’s very busy.” (CPF2).

This structural strain interacted with the next barrier teachers discussed: the absence of targeted loneliness interventions. Schools across both countries prioritize visible symptoms, such as physical disabilities, learning difficulties, or behavioral problems, over subjective feelings like loneliness. Even though some schools were “very good at looking at mental health and ensuring wellbeing, but not specifically at loneliness” (BPF1). This absence of targeted provision often left teachers feeling that pupils who were lonely were “hidden” within existing support frameworks, falling between formal categories of need and therefore receiving little direct attention.

### 3.4 Theme 4: Teachers within a multi-layered attachment ecology

The fourth theme relates to teachers placing of their role in a broader context and their perspectives on the support that significant others can provide to lonely pupils: *4a Recognizing Their Limited Influence Compared to Families and Peers*, *4b Acting as Key Connectors Across the Attachment Network*, and *4c Cultural and Contextual Barriers to Effective Support*.

#### 3.4.1 4a. Recognizing their limited influence compared to families and peers

When teachers from both contexts ranked the importance of different stakeholders in supporting lonely pupils, it became clear that teachers perceive that they have a limited role compared to parents and friends. According to one Chinese secondary school teacher, “For pupils themselves, the teacher may only account for a part of this social relationship” (CSF1). Teachers felt that, from children’s own perspective, they primarily seek validation and assistance from their immediate social circles —namely, parents and peers—while the teacher’s role remains vague or secondary. This secondary role becomes more prominent only when there are gaps or issues within the family or peer support systems. One secondary school teacher from England articulated this shift: “I think then if you have got that in place, the teacher might be less important. Whereas when there’s an issue maybe with family or friends, then the teacher becomes more important to that child” (BSF2). Teachers concluded several reasons behind the perceived hierarchy of support, which included: (1) The temporary interactions between teachers and pupils limit the depth of connection that can be formed compared to the more enduring relationships children have with family and friends: “The nature of secondary schools being much larger places with lots more children in… that changes a lot, is quite fluid within that” (BSF2); “Since pupils interact with multiple teachers daily, any single teacher can only play a limited role within the limited time they have” (CSF1); (2) Teacher support is often seen as a professional obligation, whereas support from parents and friends is perceived as voluntary and driven by genuine care and empathy: “It is teachers’ job, but others [provide support] because they want to, [out of care and concern]” (BSM1).

Parents were consistently ranked as the most crucial support system for providing fundamental love and emotional security: “Home will always be No. 1” (BSM1). One English primary school teacher highlighted the significance of parental awareness, saying, “She’s quite shy and inward looking. And I had not really noticed [her loneliness] and I think that can be quite dangerous. And she was lucky that her mom did notice and [intervened]. But obviously not all children have that” (BPF3). Friends also play a vital role by offering consistent companionship: “Your friends move through the school with you” (BPF3). However, teachers acknowledged the inherent variability and unpredictability in peer relationships, highlighted that “the quality of the peer group is uncontrollable” (CPF2). As one teacher observed, “Every pupil has his or her own personality. It is not possible to take care of their friends’ feelings all the time” (CSF2). Cultural contrasts also shape support dynamics, with teachers in England emphasizing sibling support (BPF3), while Chinese teachers focus more on the involvement of extended family members, like grandparents (CPM1), and community support (CPF1).

#### 3.4.2 4b. Acting as key connectors across the attachment network

As one Chinese secondary school teacher articulated, “a good classroom teacher cannot make loneliness disappear completely but can shorten the gap” (CSM1). Teachers play an irreplaceable role in collaborating with various stakeholders to support lonely pupils. Besides fostering peer communication in the classroom, teachers can facilitate open dialogues with parents, many parents are unaware of their children’s social needs and may harbor stigmas and misconceptions surrounding their child’s loneliness. For instance, one teacher in China pointed out, “Parents are often unwilling to accept [their child’s loneliness]. It indicates a failure of their parenting, and they do not want to admit this fact. Then some parents will make only superficial changes” (CSF3). In China, home visits are a special strategy employed to understand pupils’ lives better. These visits allow teachers to indirectly understand pupils’ family backgrounds and communicate with parents about their child’s behavior and potential influences. While in England, parents are more likely to engage with the school environment. For example, engagement is often facilitated through structured school events like parents’ evenings, open days, and official school communication platforms (e.g., emails or newsletters), rather than through visits to the family home.

In addition, teachers serve as liaisons with appropriate support services within the school or externally, building a network to identify pupils experiencing long-term loneliness and referring them to appropriate support services. As one English secondary school teacher emphasized: “It has to be more than just me as an individual to be able to identify… also being aware of where to signpost pupils to for further help, whether that be like within the school, we’d like [to know] the safeguarding team or looking at external support that can actually help pupils” (BSF1). This referral process is not just about directing pupils to the right services; it also helps to bridge the gap between pupils and those external supports. Often, pupils may build up defenses or feel a lack of trust toward unfamiliar services. A teacher’s involvement can help to lower these barriers, making it easier for pupils to accept the support they need. As one Chinese secondary school teacher expressed “They do not know what kind of person they are facing. So, they do not have such a high level of trust” (CSF1).

#### 3.4.3 4c. Cultural and contextual barriers to effective support

Teachers working in less developed regions, such as rural areas in China, emphasized the barrier of “no tailored approaches to suit this specific context” (CPM1). As the teacher noted, “we rely more on the experience of schools in more developed regions. Well, we imitated others’ work. However, we encounter a problem that the children in urban areas are different from ours” (CPM1). Cultural differences must also be considered when learning from the experiences of schools in more developed countries. One teacher shared her thoughts on the translation of educational terms (i.e., “positive discipline”) in the Chinese context: “I think that translation is very misleading and makes people feel confused. I do not like the term ‘discipline’. If I had just seen its name when we attended that training together, I would have rejected it” (CPF3). The term “discipline” may carry certain connotations in Chinese that differ from its intended meaning in the original context.

Moreover, in interviews with Chinese teachers, it became evident that the Chinese term for “loneliness” (*gudu*) encompasses a broader spectrum of meanings than its English counterpart, “loneliness.” One Chinese teacher noted, “There will not be too much loneliness in school. I think especially in school, in such an environment, pupils are always surrounded by many people” (CSF1). In Chinese, *gudu* can refer both to the physical state of being alone and the emotional experience of loneliness. Unlike in English, where “loneliness” often carries a predominantly negative connotation, *gudu* can sometimes be a neutral or positive experience of solitude, depending on the context. As this teacher remarked, “Loneliness (*gudu*) itself is not necessarily all negative. I suddenly realized that some pupils may think it’s good and enjoy their lonely time” (CSF1). This nuanced understanding of *gudu* highlights a significant barrier for teachers: translation misinterpretations can lead to a misalignment in how loneliness is perceived; teachers may overlook the negative aspects of a pupil’s isolation or fail to identify a potential indicator of deeper issues. Additionally, the resources available to them often do not reflect the cultural and contextual realities of their environment, making it difficult to provide the necessary support.

## 4 Discussion

Through an in-depth exploration of teachers’ lived experiences with pupils experiencing loneliness, the current study sheds light on the support strategies that teachers have implemented and identifies areas in need of further development. This study provides an important perspective, that was not available before, to address this critical issue—youth loneliness in schools. Teachers openly recognized the challenges they face when trying to address loneliness, yet expressed their willingness to support lonely pupils and emphasized their potential as key intermediaries between pupils, parents, and other stakeholders. They highlighted the pressing need for more structured support and targeted training to better equip them. Together, the findings from the interviews encompass three key areas: (1) the barriers that hinder teachers’ efforts, (2) the existing gaps in teacher training related to loneliness, and (3) school-based interventions as viewed through both teacher and cross-cultural perspectives. In the following discussion, we will discuss and explore each of these key aspects in greater detail.

### 4.1 Empowering teachers to combat pupil loneliness: suggestions for teacher training

**Figure 2:**
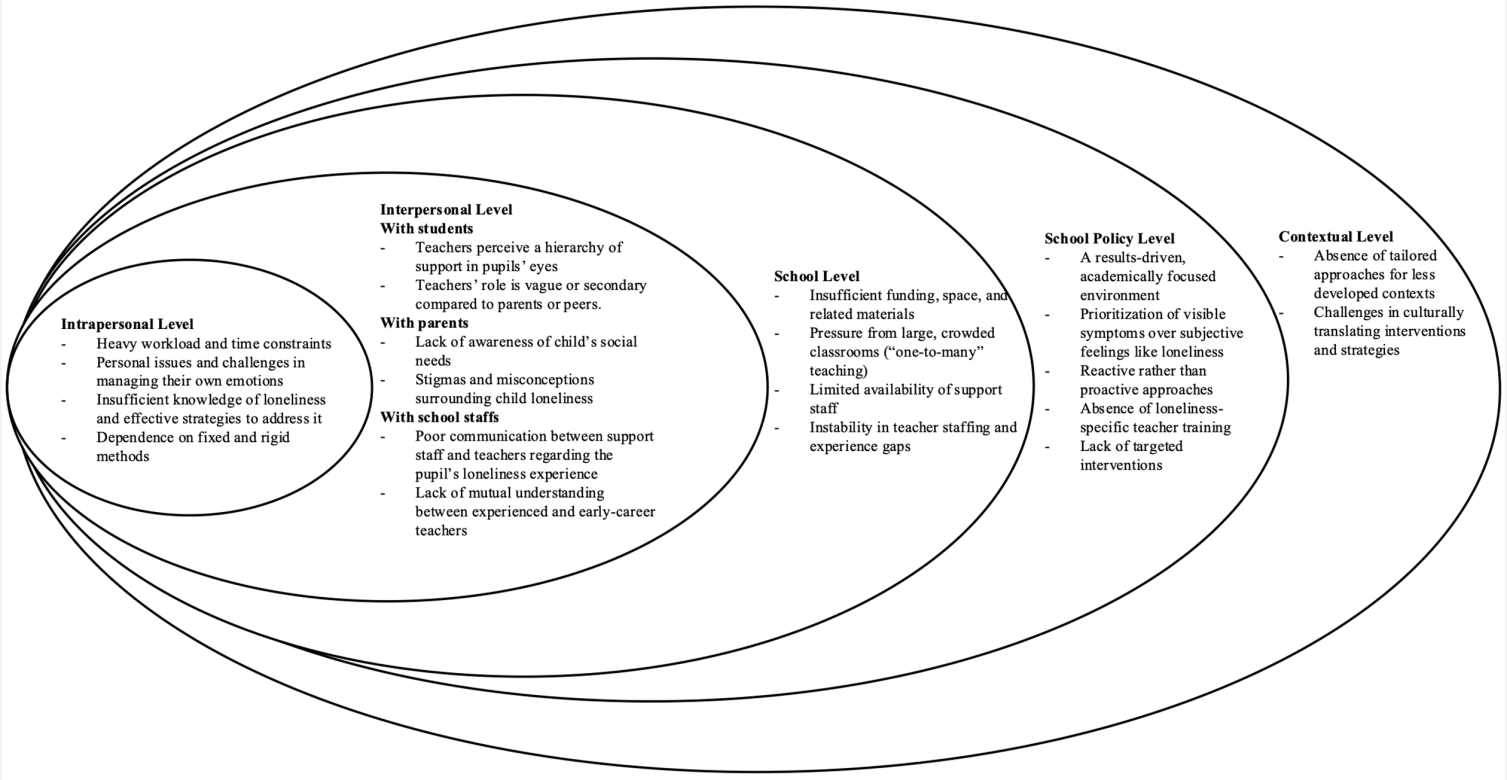
Teacher support barriers.

Teachers shared strategies they have found effective in supporting pupils experiencing loneliness, stemming from their direct classroom experiences. While those methods were noted as helpful, they also pointed out a gap in formal knowledge and training about loneliness, which presents a challenge in accurately identifying and addressing loneliness. Teachers highlighted the barriers they encounter in their efforts, often feeling that their actions fall short without a deeper understanding of loneliness and more structured support systems. Those barriers can be understood through the lens of Bronfenbrenner’s ecological model (Bronfenbrenner, 1979), which allows us to categorize the challenges across different layers of influence, from the individual to the broader societal context (see Figure 2).

**Figure 3:**
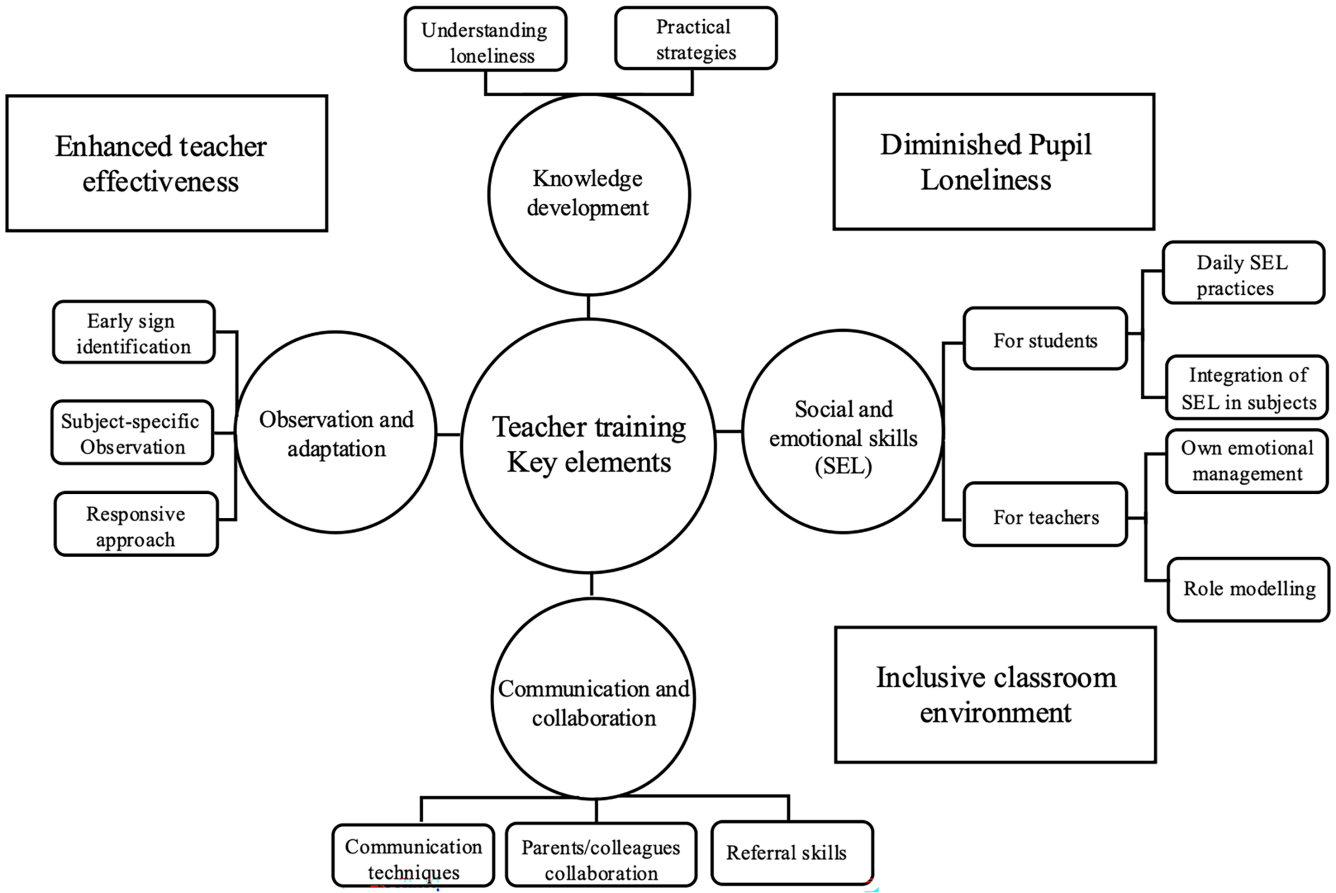
Key elements and proposed outcome of teacher training.

Given the multitude of barriers, relying solely on individual teacher practices is not enough to provide the necessary support for lonely pupils. The challenges faced by teachers underscore the need for a more comprehensive and systematic approach to teacher training. While mental health training for teachers has been well-documented and shown to be promising (e.g., Anderson et al., 2019; Banwell et al., 2023), there remains a noticeable gap in training specifically designed to address loneliness. Building on the insights and suggestions from teachers, we propose four core elements that should be integrated into teacher training programs aimed at addressing pupil loneliness (see Figure 3):

#### 4.1.1 Knowledge development

Teachers need training on the nature of loneliness, its causes, and common manifestations in pupils. That training will equip them with the knowledge to effectively introduce and discuss loneliness within the classroom setting. Teachers will also learn how to translate theoretical understanding into practical strategies, such as developing lesson plans, activities, and interventions specifically designed to address loneliness among pupils. Basic knowledge and literacy about loneliness are essential components for teachers to implement and support the delivery of high-quality prevention and intervention programs, thereby enhancing the effectiveness of school-based initiatives. Research on broader mental health support in schools also underscored the importance of equipping teachers with related knowledge, demonstrating their role in fostering positive student outcomes and reducing risks associated with unaddressed emotional struggles (Fortier et al., 2017).

#### 4.1.2 Social and emotional skills

For pupils, teachers should be trained to incorporate social–emotional learning (SEL) activities into daily classroom routines and seamlessly integrate SEL into subject-specific teaching (Ferreira et al., 2020). Research has identified social and emotional training as one of the most effective intervention strategies for reducing loneliness among youth (Eccles and Qualter, 2021). A core focus of such training should be on enhancing peer interactions, fostering group dynamics, and promoting a cohesive classroom environment. Equally important is the focus on teachers’ own emotional wellbeing and management, as these significantly influence students’ emotional and social experiences (Schonert-Reichl, 2017). Effective training should emphasize reducing emotional distance and fostering empathy. It will also cover techniques for regulating their emotions, particularly when dealing with pupils who are lonely or struggling socially. Teachers will also learn to serve as role models for emotional regulation and appropriate social behavior.

#### 4.1.3 Communication and collaboration

Teachers’ communication skills should be enhanced through systematic training in effective communication techniques, both verbal and non-verbal, as well as strategies for working collaboratively with parents, colleagues, and external support networks. Teachers should be trained on when and how to refer pupils to additional support services, ensuring that pupils who need more specialized assistance receive it promptly and with minimal defensiveness. Although the need was highlighted by teachers during our interviews, a recent review by Ohrt et al. (2020), based on existing evidence related to mental health, showed that teacher education programs often overlook this practical aspect. Addressing this shortfall would empower teachers to act confidently, but also ensure that pupils experiencing loneliness or other social challenges are connected with appropriate resources in a timely and empathetic manner.

#### 4.1.4 Observation and adaptation

Teachers can develop observation skills to identify signs of loneliness, which are often subtle and easily overlooked. By being attentive and proactive, teachers can serve as active observers (Rothì et al., 2008), identifying pupils who may be struggling with loneliness, but also raising their visibility within the school as part of a supportive system. Importantly, teachers should be taught to recognize how loneliness may manifest differently depending on the subject matter. This sensitivity will enable teachers to identify loneliness more accurately and implement subject-specific interventions tailored to the individual needs of each pupil.

The implementation of such training should involve interactive workshops and case studies, enabling teachers to apply strategies in real-life scenarios and think critically and adaptively when handling various situations involving loneliness. Three key outcomes would come from such training: enhanced teacher effectiveness in identifying and supporting lonely pupils; contribution to the creation of a more supportive, inclusive, and emotionally safe classroom environment; and reduction in pupil loneliness by fostering better emotional and social outcomes and cultivating a stronger sense of belonging among pupils. Figure 3 presents a draft framework outlining those key elements, how they can be achieved, and the anticipated outcomes. This is a brief outline based on findings from our study; future studies could delve deeper into the layers of barriers teachers face and gather more insights from teachers. Following this, it would be useful to engage in co-production with various stakeholders to develop a targeted and comprehensive training model for reducing adolescent loneliness, and then undergo pilot testing.

### 4.2 Rethinking school-based intervention based on teachers’ perspective

Children and adolescents spend a substantial amount of their day in school. Thus school, and especially teachers, have an important role in preventing and/or intervening to help children and adolescents who suffer from loneliness. By gathering teachers’ insights, our study fills a critical part of this picture—practical considerations involved in this process—that has been missing until now. The teacher insights prompt us to rethink and ground school-based interventions in more realistic and applicable ways, ensuring they are more effective and attuned to the actual needs and circumstances of both pupils and teachers.

Teachers in our study recognized the negative impacts of loneliness and expressed a strong willingness to support pupils experiencing it, which is promising. However, they noted a significant gap: their schools currently lack targeted intervention program specifically addressing loneliness. Often, schools do not prioritize the issue of loneliness or chronic loneliness sufficiently. Notably, when discussing potential interventions, teachers frequently linked loneliness to mental health, even without prompting. This connection suggests that, rather than creating isolated programs solely for loneliness, it may be more feasible to integrate loneliness-specific interventions within the broader mental health framework, such as more established mental health literacy programs, social–emotional learning interventions or even school transition interventions (recognizing the increase in loneliness during transition periods), ensuring they fit seamlessly within the existing school infrastructure. An example is the Promoting Alternative Thinking Strategies (PATHS) program, a school-based universal curriculum that includes lessons on loneliness and has been found to significantly reduce loneliness in children (Hennessey et al., 2021). Future research should focus on developing such integrated programs, exploring their long-term impacts, particularly in diverse school settings, and assessing their effectiveness across different age groups, including adolescents.

Moreover, teachers described their role as being stuck in a reactive mode, akin to firefighters addressing immediate issues rather than preventing them. They emphasized the importance of proactive prevention—building a whole-school or whole-class supportive climate that fosters successful reconnections and actively prevents transient loneliness from becoming prolonged and chronic (Jefferson et al., 2023b). However, they also expressed concerns about their role being viewed as secondary to that of parents and peers. While teachers serve as crucial sources of guidance and support, they often intervene when there are gaps in family or peer support systems. From teachers’ perspectives, pupils often view teacher-pupil relationship as more formal and less emotionally intimate compared to their relationships with parents and peers. Teachers are seen more as authority figures than as confidants, which poses a significant obstacle to fostering a truly inclusive and supportive school climate. This indicates there is still a wall or emotional distance built by pupils or teachers themselves, which stand as a barrier to foster a truly inclusive and belonging school climate. Those concerns have been highlighted in the recommended teacher training programs, and highlight the need for ongoing efforts and additional measures to bridge this emotional gap and enhance the overall school climate.

### 4.3 How culture might affect teacher support strategies

Our study revealed the nuanced cultural differences in the strategies teachers use to support pupils are what stand out. In China, it is common for teachers to conduct home visits, a practice that extends the school’s influence into the home environment, thereby reinforcing the connection between the microsystems of school and family. This practice aligns with Confucian values and collectivism that prioritize building personal connections and maintaining social harmony. It emphasizes the role of teachers as educators, but also as integral parts of the pupils’ broader social support network. In England, however, teachers often adopt a more practical perspective, focusing on interventions that address pupils’ immediate social needs, reflecting a more outcome-oriented view. The contrast between these approaches—long-term, community-focused in China versus immediate, individual-focused in England—highlights the need for culturally sensitive interventions. Practically, this suggests the value of context-specific toolkits: in China, guidance could support teachers in conducting home visits in ways that are developmentally appropriate, emotionally sensitive, and focused on listening rather than surveillance or academic monitoring; in England, structured classroom-based strategies, such as peer-inclusion routines, staff-led social check-ins, and clear referral pathways, may help teachers respond to loneliness within time-limited and accountability-driven school environments.

Culturally sensitive interventions highlight the importance of adapting universal elements of effective practices to fit diverse cultural contexts. Such an intervention approach ensures cultural relevance and respect of local norms, which in turn makes the guidance provided to teachers and other implementers more grounded and effective. Moreover, cultural sensitivity also needs to exist in the exchange and reference of ideas and practices across different cultural contexts. Teachers, particularly in China, emphasized that strategies from Western countries cannot be directly transplanted into their context without careful adaptation. An example of that challenge is the translation of “loneliness” and the Chinese term *gudu*. The boarder meaning of *gudu*, encompassing both solitude and loneliness, may lead to misunderstanding among teachers and impact the strategies they adopt. It could also affect the use of loneliness scales, which are often developed in English-speaking countries and may be directly translated into other languages, without cultural adaptation. This direct translation risks failing to capture the intended meaning and emotional experiences of individuals within their cultural context. Therefore, there is a pressing need to remain culturally sensitive and to develop or adopt tools and strategies that accurately reflect the lived experiences and cultural meanings of the populations being studied. This involves more than just translating words—it requires a deep and thoughtful understanding of the cultural context. By doing that, teachers and researchers can ensure that the interventions and assessments they employ are truly effective and relevant within the specific cultural setting they are intended to serve.

### 4.4 Strengths and limitations

Our study examined teachers’ perspectives and strategies on loneliness and we have put forward practical recommendations for teacher training and several strategies for school-based interventions to address pupil loneliness. To summarize, the practical suggestions are as follows: (1) integrating loneliness-specific interventions within a broader mental health framework; (2) fostering proactive prevention by building a whole-school/class supportive climate that reduces emotional distance between pupils and teachers; (3) developing adaptable, universal frameworks for addressing loneliness, ensuring that interventions can be tailored to different cultural contexts; and (4) advocating for a more comprehensive and systematic approach to teacher training related to identifying, monitoring, and responding to loneliness in pupils. These recommendations highlight the importance of a coordinated, culturally responsive, and well-supported approach to addressing loneliness in schools.

While those insights provide valuable guidance, our study has several limitations, which offer direction for future research. To gain a more comprehensive understanding of loneliness and the potential for effective interventions, future research should involve a more diverse and larger group of participants. This could include a wider range of teachers, but also other key stakeholders such as school support staff (e.g., teaching assistants and lunchtime staff who might have more insight into pupils social interactions outside the classroom), parents/carers, and other pupils. This holistic approach would allow for the identification of additional barriers and solutions across various layers of influence, such as family dynamics, community involvement, and societal norms, and would help to understand how these layers interact to shape loneliness and the effectiveness of interventions.

Additionally, while we interviewed teachers from both primary and secondary schools, our analysis did not distinctly differentiate between the unique challenges and needs at these educational levels. This decision was made to avoid over-interpretation based on a limited number of participants within each subgroup, particularly given the study’s primary focus on cross-cultural comparison. Disaggregating findings simultaneously by national context and school level would have risked fragmenting the analysis and reducing analytic coherence. Nevertheless, we acknowledge that teacher–pupil relationships, opportunities for emotional attunement, and perceived barriers to intervention can differ between primary and secondary education. Future research with larger and more stratified samples should examine how experiences of supporting lonely pupils vary across educational levels. Such work would not only deepen theoretical understanding of age-related relational dynamics in schools, but also enhance policy relevance by informing developmentally tailored training, resource allocation, and intervention strategies across different stages of schooling.

Finally, while our study underscored the importance of cultural sensitivity in loneliness interventions, further research is needed to explore and refine culturally sensitive methods in greater depth. This involves identifying universal elements of effective interventions that can be applied across contexts, as well as determining which aspects require cultural adaptation. Understanding the specific cultural contexts in which pupils live and learning to respect and incorporate local values and practices into interventions is crucial for ensuring their success and relevance.

## 5 Conclusion

The current study explored how teachers —one of the major attachment figures in young people’s lives— from England and China understand and respond to pupil loneliness, highlighting their central yet constrained role within school settings. Across both contexts, teachers expressed a clear willingness to support lonely pupils, but also identified multiple barriers that limited their capacity to do so effectively. Organized through Bronfenbrenner’s ecological model, these barriers ranged from intrapersonal challenges, such as uncertainty in identifying loneliness, to external constraints like limited school facilities and result-driven school policies. As a result, support was often reactive and focused on more visible or acute cases, rather than embedded within preventative, whole-school practices. Drawing on those insights, we developed a series of recommendations aimed at enhancing teacher training and refining school-based interventions to better tackle pupil loneliness.

The cross-cultural comparison reveals both shared challenges and context-specific strategies. Teachers in England tended to prioritize practical, time-efficient interventions that addressed pupils’ immediate social needs, while teachers in China more frequently described relationally expansive practices—such as home visits—that extend support beyond the classroom and strengthen school–family connections. Differences in how loneliness is linguistically and culturally understood, particularly the broader meanings associated with *gudu* in Chinese, further shaped teachers’ interpretations and responses. Taken together, these findings underscore the need for culturally sensitive, system-level approaches to addressing pupil loneliness.

## Data Availability

The datasets presented in this study can be found in online repositories. The names of the repository/repositories and accession number(s) can be found in the article/Supplementary material.
