# Supplementary material for: “A small change can make a huge difference”: teachers’ perceived roles, barriers, and strategies in tackling loneliness in schools
Source: Front Psychol. 2026 Feb 23;17:1766329. doi: 10.3389/fpsyg.2026.1766329 (PMC12968173; doi:10.3389/fpsyg.2026.1766329)
Supplement: Supplementary file 1 [file Supplementary_file_1.docx]

**Interview Guide**

**Pre interview questions**

Is there anything you want to ask me before we start?

Within each section of questions prompts such as – can you tell me more about ... or what

was that like ... will be used to extend answers if that is deemed appropriate for that

participant.

**Section 1 – General Information**

First of all, I would like to gather some general information about you.... 1.1 What is your

role in school?

a) What subject(s) do you teach?

b) What grade(s) do you teach? (age range)

c) Can you give me a brief summary of the pupils that you teach in terms of (e.g., number,

gender proportion, ethnicity, socioeconomic status.)

**Section 2- Perceived Competence Towards Loneliness**

**2.1 How confident do you feel that you are able to identify lonely students in a classroom?**

2.2 How good effective do you think you are at helping a lonely student?

a) What skills do you have that could help a lonely student?

b) What skills would you like to acquire which might help lonely students?

c) Are there any barriers or facilitators in your school that support students in reporting

loneliness or support you in assisting lonely students?

**Section 3- Attitudes Towards Teacher-Student Relationships**

3.1 How would you describe a good teacher-student relationships? (e.g., closeness, conflict, dependency)

3.2 What do you think a supportive classroom looks like? (e.g., academic

support, emotional support)

3.3 What types of behaviours, support, or teacher-student relationships do you think would be appropriate with a lonely student?

3.4 Do you think the length of time you have known a student influences the quality of the student-teacher relationship?

a) Do you think this influences students’ loneliness?

b) What about over primary-secondary school transition?

**Section 4- Loneliness Intervention in School Settings**

4.1 Do you think it is important to help students avoid loneliness?

a) Do you think it is important to help students manage loneliness?

b) Do you think it is important to help students prevent loneliness?

4.2 Do you think that the teacher has a key role in supporting lonely students in the school

context?

a) If not, who do you think is responsible for supporting students who are lonely?

b) What about sort of outside school, in the social context, if you ranked the importance of supporting lonely students from parents, teachers, friends, and classmates, how would you rank them?

4.3 Is the teacher’s role clearly or vaguely defined towards lonely students in your school?

4.4 Are you willing to be involved in loneliness intervention programmes outside of your

usual teaching duties?

a) Can you suggest some possible reasons or factors for why or why not?

4.5 Have you attended any professional development training about supporting students with loneliness?

4.6 What does your school do to prevent loneliness?

a) How effective do you think it is?

b) What do you think could make these interventions more effective?

c) If your school does nothing, what do you think they could do to prevent loneliness?

4.7 Does your school offer any training modules to avoid/manage/prevent students’ loneliness or develop students’ social and emotional skills? Do you think it is necessary?

**Post interview questions**

What kind of advice or tips would you like to add?

**Following the interview**

Thank the teacher for participating in the interview and read out the debrief sheet.
